# Understanding key factors contributing to mental health challenges among pediatric nurses: a systematic review

**DOI:** 10.3389/fpubh.2025.1480024

**Published:** 2025-02-26

**Authors:** Godfrey Mbaabu Limungi, Mesmar Amer, Mohammed Elmadani, Klara Simon, Osama Hamad, Eva Horvath, Patty Livia, Mate Orsolya

**Affiliations:** University of Pécs, Pécs, Hungary

**Keywords:** mental health challenges, pediatric nurses, mental-wellbeing, key factors, pediatric units

## Abstract

**Background:**

The mental health and wellbeing of nurses, particularly pediatric nurses, have garnered attention due to the increased risk of mental health challenges associated with their demanding profession. These nurses are especially vulnerable, yet their mental wellbeing is often understudied.

**Objective:**

This systematic review aims to identify and analyze key factors associated with mental health challenges among pediatric nurses and explore how these factors interact to influence their wellbeing.

**Methodology:**

The review protocol was registered in PROSPERO (CRD42024553062) and adhered to PRISMA guidelines. A comprehensive search was conducted across six databases: PubMed Scopus, CINAHL, Web of Science, Medline, and Embase. Eligible studies included both qualitative and quantitative studies that examined factors linked to mental health challenges among pediatric nurses. The quality of the studies was appraised using the Mixed Methods Appraisal Tool (MMAT). Data extraction and synthesis involved qualitative content analysis to identify key factors.

**Results:**

Five studies from China, Turkey, Greece, Canada, and Saudi Arabia were included. The key factors identified were high workload, poor work environment, limited resources, and strained interpersonal relationships, lack of support, irregular shift patterns, demanding roles, and financial strain. These factors were significantly associated with increased stress, burnout, anxiety, and depression among pediatric nurses. The interaction of these factors created a complex web influencing their mental health, with supportive work environments and adequate financial compensation mitigating some negative effects.

**Conclusion:**

This systematic review identifies high workload, poor work environment, limited resources, and strained interpersonal relationships, lack of support, irregular shift patterns, demanding roles, and financial strain as key factors impacting the mental wellbeing of pediatric nurses. These factors interact to exacerbate stress, burnout, anxiety, and depression. Effective interventions should include manageable nurse-to-patient ratios, adequate resource allocation, fostering a supportive work culture, flexible scheduling, targeted support for senior nurses, and improved financial compensation.

## Introduction

In recent years, the mental health and wellbeing of nurses have garnered attention due to the increased risk of mental health challenges associated with their demanding profession. Among nursing specialties, pediatric nurses face unique and intensified challenges that make them particularly vulnerable to stress, burnout, anxiety, and depression. Unlike nurses caring for adult patients, pediatric nurses often deal with emotionally charged situations involving vulnerable children, end-of-life care for young patients, critically ill pediatric patients, and the complexities of family-centered care. They must not only provide nursing care to the children but also support anxious, grieving, or highly expectant parents, which can introduce a significant emotional and ethical strain. The distress of witnessing suffering in children, coupled with moral dilemmas and high parental expectations, can contribute to an overwhelming work environment (1, 2, 31).

These stressors, compounded by the physically and mentally demanding nature of pediatric nursing, have been linked to a higher prevalence of mental health issues in this group (2, 31, 32). Research has consistently shown high levels of stress, burnout, anxiety, and depression among pediatric nurses (2–7, 32). Stress, as defined by the World Health Organization (WHO), occurs when an individual perceives that demands exceed their ability to cope, leading to physiological and psychological strain (8).

According to Nkyi and Baaba (33), stress arises when environmental challenges exceed an individual's physical and mental resilience. Pediatric nurses frequently encounter Secondary Traumatic Stress (STS), a condition arising from continuous exposure to patients' suffering experiences. Studies have reported moderate to severe STS in these nurses, with prevalence rate ranging between 40% and 78.2% (2, 9–11). For instance, Yehene et al. (2) found that 77.8% of pediatric nurses experienced moderate to severe levels of STS. Similarly, Berger et al. (9) reported a rate of 78.2%, while Günüşen et al. (10) and Kellogg et al. (11) observed prevalence rates of 40.6% and 50.3% respectively.

Burnout, as described by World Health Organization (12), is a syndrome resulting from chronic workplace stress that has not been successfully managed. It is characterized by emotional exhaustion, depersonalization, and reduced professional efficacy. Emotional exhaustion rates among pediatric nurses range from 30.77% to 73.70% (3, 13, 14). Anxiety and depression are also highly prevalent among peadiatric nurses. According to the Diagnostic and Statistical Manual of Mental Disoders (DSM-5), anxiety disoders involve excessive worry, restlessness, and physiological symptoms such as tachychardia, while depression is characterized by persistent sadness, fatigue, cognitive imparements and lack of interest (15). Maharaj et al. (16) reported anxiety and depression prevalence rates of 41.2% and 32.4%, respectively. In an Indian tertiary hospital, Shajan and Nisha (5) observed anxiety in 40% of nurses and depression in 35.8%. Similarly, Ghawadra et al. (6) found that 39.3% of nurses in a Malaysian teaching hospital experienced anxiety, and 18.8% suffered from depression. A study across 34 hospitals in China, reported even higher rates −44.6% for anxiety and 50.4% for depression (7).

The consequences of these mental health challenges extend beyond individual wellbeing to include professional performance and organizational outcomes (16–18). These mental health issues have been linked to increased intent to resign, physiological diseases, reduced quality of care to the patients, increased maladaptive behaviors, and financial implications to the health institutions (16–20). Despite the critical nature and profound impact of these mental health challenges on pediatric nurses, their mental health and wellbeing remain underexplored in research (1), leaving critical gaps in understanding the factors contributing to their distress. Additionally, the COVID-19 pandemic has exacerbated mental health conditions across nursing populations including pediatric nurses, further highlighting the need for targeted interventions. Therefore, this systematic review aims to address these gaps by thoroughly examining these factors, providing insights for practice initiatives and future research in this crucial area.

## Methodology

### Research question

What are the key factors strongly associated with mental health challenges among pediatric nurses, and how do these factors interact and collectively influence their overall mental wellbeing?

### Protocol and registration

The review protocol was registered in the international register of the Prospective Register for Systematic Reviews (PROSPERO) on June 11, 2024, with registration number (CRD42024553062). The review adhered to the standards set by the Preferred Reporting Item for Systematic Reviews and Meta-Analyses (PRISMA) statement (21).

### Eligibility criteria

The review included all studies involving quantitative, qualitative, or mixed methods, published in English in peer-reviewed journals and focusing on factors associated with mental health challenges, such as workload, patient acuity, shift patterns, workplace support, coping mechanisms, and personal characteristics. Studies that focused on pediatric nurses as the study population or subpopulation from any specialization in any pediatric setting (e.g., pediatric general wards, pediatric intensive care unit, newborn unit, oncology, renal unit, and pediatric emergency department) were included. Discussion papers, dissertations, theses, commentaries, editorials, systematic reviews, scoping reviews, meta-analyses, and literature reviews were excluded from this review. Dissertations and theses were excluded because they may compromise the reliability of the findings, as they often undergo limited peer review. Studies solely focusing on interventions or treatments for mental health challenges without exploring associated factors, studies with a low-quality evidence (e.g., case reports or case series, and those with insufficient data or unclear methodology were also excluded. Low-quality evidence was defined based on factors which included study design, sample size, and lack of transparency in the methodology.

### Databases and search strategies

Two research team members (GML and MA) independently conducted a comprehensive search across six databases according to eligibility criteria. The included databases were PubMed, Scopus, CINAHL, Web of Science, Medline, and Embase. The search was done on different days in the 2^nd^ week of June 2024, without any restrictions on publication date (from inception to 14^th^ of June 2024) ensuring comprehensive inclusion. The search strategy included (“mental health challenges” OR “mental health issues” OR “psychological stress” OR “burnout” OR “anxiety” OR “depression”) AND (“pediatric nurses” OR “children's nurses” OR “pediatric nursing staff”) AND (“factors” OR “contributing factors” OR “causes” OR “determinants”). Notably, the search did not include the alternate spelling “pediatric nurses” which we acknowledge as a limitation, as it may have led to the unintentional exclusion of some relevant studies. Articles from these databases included pediatric nurses either as a sample population or a subset of the larger study population. In this review, pediatric nurses were defined as any nurse working in a pediatric unit, irrespective of whether they had specialized training in pediatric nursing.

### Selection of sources of evidence

**Figure 1:**
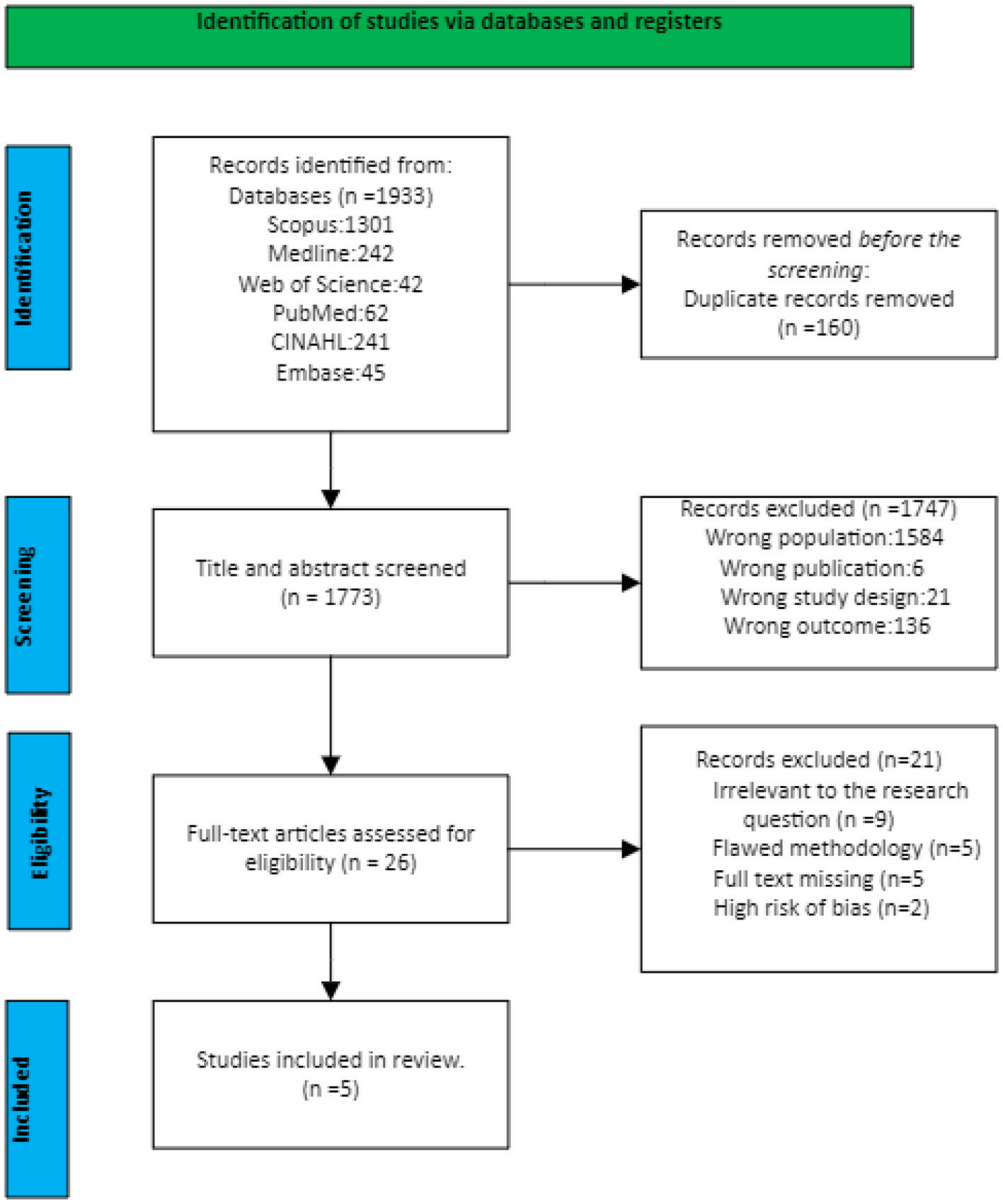
PRISMA flow diagram of study selection.

After retrieving the articles from the databases, they were uploaded to the Rayyan software (22) and duplicates were removed before screening was performed by three research team members (GML, MA, and ME). Two reviewers from the research team (GML and MA) evaluated independently all the papers by titles and abstracts to determine if they met the selection criteria through a blind process. Any disagreement was resolved by the third reviewer (ME). The selected articles were subsequently screened as full-text articles for inclusion again involving a blind process. Arising conflicts were resolved by discussion among the three reviewers (GML, MA, and ME). The selection process is depicted in Figure 1.

### Quality appraisal

An evaluation of the quality of individual studies was performed using a mixed methods appraisal tool (MMAT), which is a helpful tool for evaluating the quality of quantitative, qualitative, and mixed-methods research (23). MMAT was selected due to its unique flexibility in evaluating studies across multiple methodologies. The appraisal process considered various dimension of bias including selection bias, measurement bias, reporting bias, publication bias and risk for self-reporting bias. While MMAT provided a comprehensive framework, some challenges arose during the evaluation process, particularly when interpreting risk of bias in studies with insufficient reporting. To address these challenges, a third reviewer was involved to resolve any discrepancies through discussion ensuring consistency in evaluations. Each study was categorized into one of the three levels of bias: “low risk of bias,” “some concern of bias,” and “high level of bias,” based on specific study elements such as sample size, data analysis, and clear reporting of the methodology. For example, studies with sufficient sample size, well-elaborated methodology and comprehensive data analysis and transparency in reporting were categorized as “low risk of bias.” Those that had insufficient sample size, and possible risk for self-reporting bias but still providing valuable insight were categorized as “some concern of bias,” while those with insufficient sample size, unelaborated reported methodology, inappropriate statistical analysis or high likelihood of reporting bias were categorized as “high-level bias.” To ensure the accuracy of the evaluation, two research team members (GML and MA) independently assessed the risk of bias and quality of each study. In case of disparity, they consulted the third research team member (ME) to reconcile any difference. Table 1, provides a summary of distribution of studies across these bias categories showing how many studies were classified as “low risk of bias,” “some concern of bias,” and “high level of bias.”

**Table 1:** Distribution of studies across bias categories.

**MMAT parameters**	**Low-risk bias**	**Some concerns of bias**	**High level of bias**
Strong methodology with a substantial sample size. A clear description of variables and control for confounders. Robust statistical analysis.	1		
Well-described methodology with sufficient sample size and comprehensive statistical analysis.	1		
Clear methodology, large sample size, valid measurement tools, and proper statistical analysis conducted	1		
Small sample size, clearly described methods, possible risk of self-reporting bias present.		2	
Small sample size, unelaborated methodology			1
Sufficient data, inappropriate data analysis, unclear reporting methodology			1
**Total studies**	**3**	**2**	**2**

### Data charting process

This systematic review used a data extraction table, developed by the principal researcher to extract data from the included studies. Before data extraction, this tool was pretested, and the research team agreed that it could capture the intended data. The data that were extracted included study details (authors, year of publication, study design, and country where the study was conducted), mental health challenges, factors associated with these challenges, description of pediatric units (e.g., pediatric general wards, pediatric intensive care unit, newborn unit, oncology, renal unit, pediatric emergency department) involved, significant value, conclusions, and recommendations.

### Data synthesis and analysis

Data were synthesized qualitatively through content analysis utilizing an inductive coding approach. Themes emerged directly from the data, capturing key factors strongly associated with mental health challenges among pediatric nurses and illustrating their interplay in influencing mental wellbeing. (24). To ensure reliability and validity of the content analysis process, two researchers (GML and MA) independently coded the data before discussing and reaching a final consensus on the final coding. Additionally, the second reviewer (MA) cross-checked the synthesis to ensure consistency and minimize potential bias.

## Results

This systematic review aimed to identify and analyze the key factors contributing to mental health challenges among pediatric nurses. The initial database search yielded 1,933 potential articles from which 1,773 publications were selected for consideration after removing 160 duplicates. These articles were published between 1991 and 2024, with most of the publications occurring between 2018 and 2024. A thorough title and abstract screening resulted in 1,747 publications being excluded for various reasons as illustrated in Figure 1. Of the 26 articles selected for full-text screening, 21 studies were ultimately excluded based on the established inclusion and exclusion criteria. Consequently, five studies were deemed relevant and included for data extraction and analysis. The characteristics of these studies are detailed in Table 2.

**Table 2:** Characteristics of 5 studies exploring factors contributing to mental health challenges among pediatric nurses included in the systematic review.

**References**	**Study design**	**Sample size**	**Pediatric unit**	**Country study done**
Liao et al. (1)	Descriptive cross-sectional	500	Inpatient department	China
Kartsonaki et al. (25)	Descriptive cross-sectional	75	PICU	Greece
Yildirim and Ertem (26)	Cross-sectional	316	All units	Turkey
De Almeida Vicente et al. (27)	Qualitative cross-sectional	12	Medical and Surgical	Canada
Alharbi and Alkhamshi (28)	Descriptive cross-sectional	250	All units	Saudi Arabia

All included studies (*n* = 5) utilized a cross-sectional study design with only one study specifically focusing on pediatric nurses as the subset of the larger population. These studies were conducted across five countries: China (*n* = 1), Turkey (*n* = 1), Greece (*n* = 1), Canada (*n* = 1), and Saudi Arabia (*n* = 1).

### Key factors contributing to mental health challenges among pediatric nurses

This systematic review identified several key factors significantly contributing to mental health challenges among pediatric nurses. These factors relate to work environment, workload, support system, financial income, and shift pattern as illustrated in Table 3.

**Table 3:** Factors contributing to mental health challenges among pediatric nurses.

**Mental health challenge**	**Factors**	**Evaluation (significance)**	**Conclusions drawn by the studies**
Stress (8–12 yrs of service) Anxiety and depression (4–7 yrs of service)	Time allocation and workload Work environment and resources Patient nursing and management Interpersonal relationships Negative coping	*P < * 0.001 *P < * 0.001 *P < * 0.018 *P < * 0.003 *P < * 0.05	Pediatric nurses with 4–7 and 8–12 years of service have higher levels of workplace stress and are more likely to experience anxiety and depression.
Stress Burnout	Bad working conditions Rolling shifts Not working in a department by choice Younger people	*P < * 0.001 *P < * 0.011 *P < * 0.001 *P < * 0.029	Health professionals must deal not only with stress and increased workload but also with their personal feelings. Hospital managers should be alert and implement interventions with counseling and stress reduction techniques.
Burnout	Low monthly income level Monthly Night-Shift Frequency Average Weekly Working Hours	*P* < 0.003 *P < * 0.036 *P < * 0.002	work-related variables such as “monthly number of shifts,” “monthly income level,” and “average weekly working hours” impacted nurses' professional quality of life.
Stress	Limited resources Assuming multiple and demanding roles Lack of support	Perceived stressors (“There is less equipment, less personnel, less of things that you need, and more running around and finding things, and equipment and resources… I find it more stressful” When you're working at night, you're the most senior and everyone… expect[s] you to have all the answer[s], and you have to know how to deal with the situation and sometimes it's very stressful “I don't feel like there is much support for the senior nurses.”	Challenges to care delivery were experienced as work-related stressors. Working with limited resources, a lack of support, and assuming multiple and demanding roles can result in nurses feeling powerless to provide quality care.
Stress	Previous experience Shift length Working overtime	*P < * 0.026 *P < * 0.016 *P < * 0.004	Work-related factors, such as previous experience, shift length, and working overtime were found to have a significant contribution to STS.

### Workload and time allocation

High workloads and inadequate time allocation for patient care were consistently associated with stress, anxiety, and depression among pediatric nurses. Studies conducted in Greece by Kartsonaki et al. (25) and in Turkey by Yildirim and Ertem (26) found a significant correlation (*P* < 0.001) between these factors and mental health challenges among pediatric nurses.

### Work environment and resources

The availability and quality of resources, including equipment and personnel, were major contributing factors to stress levels among pediatric nurses. Liao et al. (1) and De Almeida Vicente et al. (27) highlighted that limited resources, contributed to stress as nurses had to “go extra mile” in providing quality patient care under constrained conditions. The qualitative insights from De Almeida Vicente et al. (27) indicated that pediatric nurses experienced increased stress when lacking essential resources, emphasizing the need for better support and resources.

### Interpersonal relationships and support

Relationships with colleagues and the level of support received from the healthcare team members were pivotal in influencing mental health among these nurses. Poor interpersonal relationships and lack of support were significantly associated with high levels of stress, anxiety and depression (*P* < 0.003). De Almeida Vicente et al. (27) provided qualitative evidence that the perceived lack of support during night shifts and in high-pressure situations increased stress among senior pediatric nurses.

### Shift patterns and work hours

The frequency of night shifts, rolling hours, and extended working hours were strongly associated with stress and burnout. Studies conducted in Turkey by Yildirim and Ertem (26) and in Saudi Arabia by Alharbi and Alkhamshi (28) demonstrated a significant association between these factors and burnout among pediatric nurses (*P* < 0.002 for weekly working hours and *P* < 0.001 for rolling shifts). Pediatric nurses working longer shifts, and more frequent night shifts reported higher stress levels and burnout rates.

### Previous experience and role demand

Nurses' previous experiences, shift length and the role they were expected to fulfill, significantly impacted their mental wellbeing. Kartsonaki et al. (25) found that these factors were associated with stress (*P* < 0.026 for previous experience, and *P* < 0.0016 for shift length). The qualitative finding from the study conducted in Canada by De Almeida Vicente et al. (27) also indicated that the expectation to manage all situations independently, especially during night shifts was a major stressor.

### Financial factors

Low monthly income levels were significantly linked to stress and burnout. The study conducted in Turkey by Yildirim and Ertem (26) reported a significant association (*P* < 0.003) between low-income and mental health challenges among pediatric nurses. Financial strain added an additional layer of stress, affecting pediatric nurses' overall wellbeing.

### Interaction of factors influencing mental wellbeing among pediatric nurses

**Figure 2:**
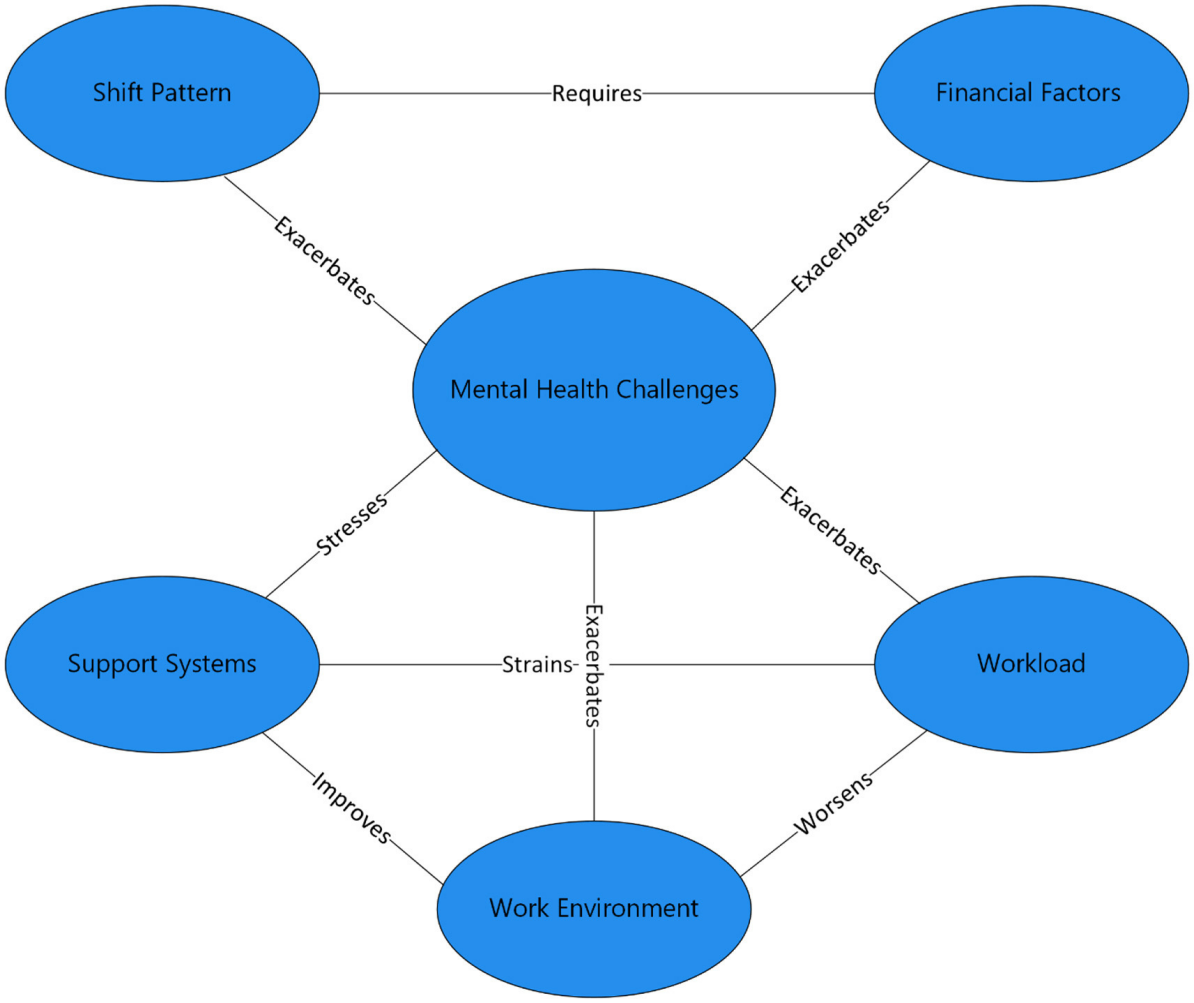
Conceptual model of factors contributing to mental health challenges among pediatric nurses.

The interaction between these factors exacerbates mental health challenges among pediatric nurses. Based on the evidence from the reviewed studies, this systematic review develops a conceptual model illustrating the interaction between these factors (Figure 2).

#### Workload and work environment

A heavy workload combined with a poor working environment leads to increased levels of stress and burnout. On the other hand, a positive work environment can reduce the negative impact of high workload. Studies by Yildirim and Ertem (26) and Alharbi and Alkhamshi (28), found significant interactions between workload and work environment, affecting stress, and burnout (*P* < 0.001, *P* < 0.018).

#### Shift patterns and financial factors

Financial needs drive pediatric nurses to work extra shifts increasing stress and burnout risk. Adequate financial compensation can reduce stress (25, 26).

#### Support systems and workload

Strong support buffers the effect of high workload, reducing stress and preventing burnout, while lack of support exacerbates the negative impact of high workload. De Almeida Vicente et al. (27) and Alharbi and Alkhamshi (28) emphasized the importance of support system in mitigating the impact of high workload on stress among pediatric nurses (*P* < 0.05).

#### Work environment and support systems

A positive work environment enhances the effectiveness of support systems, reducing stress and improving mental outcomes. On the other hand, a negative work environment diminishes the support system exacerbating mental health challenges among the pediatric nurses. According to De Almeida Vicente et al. (27) and Alharbi and Alkhamshi (28), a positive work environment with adequate resources can adequately reduce stress levels (*P* < 0.018).

## Discussion

Understanding key factors contributing to mental health challenges among pediatric nurses is crucial in developing interventions for enhancing their mental wellbeing. This review provides a comprehensive analysis of these factors, highlighting the urgency for addressing mental health challenges among pediatric nurses. Increased workload and inadequate time allocation are consistently associated with stress, burnout, anxiety and depression among pediatric nurses. Studies by Kartsonaki et al. (25) and Yildirim and Ertem (26) demonstrate a significant association between these factors and mental health challenges, highlighting the negative impact of excessive workload on psychological wellbeing. This concurs with the broader literature emphasizing the detrimental effects of increased workload on the pediatric nurse, providing insight into the need for effective workload management strategies. To prevent these issues, pediatric healthcare facilities should consider implementing policies that ensure manageable nurse-to-patient ratios and provide sufficient time for patients to be cared for and improve in intensive care units before they are transferred to general wards.

The work environment, particularly the availability and quality of resources, plays an important role in influencing stress levels among pediatric nurses. According to Liao et al. (1) and De Almeida Vicente et al. (27), resource constraints can compel nurses to exert more effort in ensuring the need for quality care of their patients, thereby exacerbating stress and burnout. This revelation is consistent with existing research suggesting that resource adequacy is a pivotal determinant of job satisfaction and mental health in pediatric nursing. To create a supportive work environment that can alleviate stress and enhance mental wellbeing among pediatric nurses, it is essential to ensure adequate resource allocation including sufficient staffing and access to necessary medical equipment.

The quality of the interpersonal relationships and the level of support from other healthcare team members remain important in impacting mental health outcomes among pediatric nurses. De Almeida Vicente et al. (27) provides qualitative evidence that a lack of support particularly during night shifts and high-pressure situations, increases stress levels among pediatric nurses. This finding is consistent with previous studies highlighting the buffering effects of a strong support system on work stress. Pediatric healthcare institutions should encourage a collaborative and supportive work culture, promoting teamwork and peer support to prevent the negative effects of stress among pediatric nurses.

Irregular work shift patterns including frequent night shifts and extended working hours are strongly associated with stress and burnout among pediatric nurses. The studies by Yildirim and Ertem (26) and Alharbi and Alkhamshi (28) demonstrates that these factors significantly contribute to burnout, emphasizing the need for better shift management to improve mental health outcomes. Documented literature shows that work shifts can disrupt circadian rhythm resulting in fatigue and psychological distress. Working during the night shift significantly affects physiological processes, raising the levels of stress hormones. This disruption often results in a state of chronic fatigue, emotional exhaustion, and cognitive anxiety, which are more prevalent among night shift workers compared to their day shift workers counterparts. Additionally, night shift work is associated with poor sleep patterns, further exacerbating these issues and impacting mental wellbeing contributing to feelings of frustration, anxiety and depression (29, 30). Ensuring the implementation of flexible scheduling and adequate resting period between shifts can help to prevent these effects and promote mental health among pediatric nurses.

The role demands of nurses significantly influence mental health among pediatric nurses. Kartsonaki et al. (25) found that this factor is associated with stress suggesting that pediatric nurses with higher role expectations face greater mental health challenges. This highlights the need for targeted support for senior pediatric nurses who may be dealing with more responsibilities. Interventions such as mentorship programs and leadership training can help equip senior nurses with the skills and support needed to manage their roles effectively and reduce stress.

Financial strain is a significant stressor for pediatric nurses, with low-income levels linked to increased stress and burnout. Yildirim reported that financial strain adds an additional layer of stress, negatively impacting the mental wellbeing of these nurses. Adequate financial compensation is critical in alleviating this stressor, suggesting that policy intervention aimed at improving the pay for pediatric nurses could have positive mental outcomes. Ensuring fair salaries and providing financial incentives can help reduce the financial burden on pediatric nurses and enhance their overall mental health and job satisfaction.

The interaction between these factors creates a complex web of influences on pediatric nurses' mental health. For instance, a heavy workload combined with a poor working environment significantly increases stress and burnout, while a positive work environment can mitigate these mental challenges. Similarly, financial stress drives pediatric nurses to work extra shifts, exacerbating stress, and burnout, whereas adequate financial compensation can reduce this need. Support systems play a vital role in buffering the effect of high workload, highlighting the importance of a supportive work culture. Based on the evidence from the reviewed studies, this systematic review develops a conceptual model illustrating the interaction between these factors. The model emphasizes the need for holistic approach in addressing mental health challenges among pediatric nurses.

### Limitations of the study

This systematic review acknowledges several limitations that may have influenced the scope and findings of this study. One notable limitation is the search strategy employed which did not include alternative spelling of pediatric (pediatric). While the review's scope was focused to ensure a targeted analysis of factors influencing mental health among pediatric nurses, this approach may have excluded some relevant studies. Additionally, this review acknowledges the paucity of studies investigating factors associated with mental health challenges among pediatric nurses as a notable limitation. Only five studies conducted across pediatric healthcare facilities in five countries were included, potentially limiting the generalization of the findings to the broader global population of pediatric nurses. The restricted number of studies and geographical scope hinders comprehensive insight into the diverse economic cultural and healthcare context in which pediatric nurses operate worldwide. Besides, the predominance of cross-sectional study designs restricts the ability to establish causal inferences regarding the relationship between workplace factors and mental health outcomes. Longitudinal studies are needed to capture evolving trends and explore causal relationships over time.

### Implication for practice

Pediatric healthcare facilities should implement policies that ensure manageable nurse-to-patient ratios can reduce high workload, which significantly contribute to stress and burnout among pediatric nurses. Besides, enhancing the work environment by providing adequate resources and necessary medical equipment can reduce the strain on nurses, allowing them to perform their nursing duties more efficiently and with less stress. Furthermore, improving financial compensation and offering financial incentives to pediatric nurses can reduce financial strain, further enhancing job satisfaction and mental health.

However, in low resource setting where staffing shortages may persist, alternative strategies such as team care models and optimizing workflow efficiency can help reduce excessive workload pressures. While financial constraints may limit adequate resources, redistributing available resources more efficiently, securing donor support and advocating for policy changes can enhance working condition. In addition, fostering a supportive and collaborative work culture through team building and peer support programs is a low cost, high impact intervention that is useful in preventing the effects of poor interpersonal relationships and lack of support. Flexible scheduling and adequate resting period between shifts are essential to combat the adverse effects of irregular shifts on nurses' mental health. Implementing mentorship programs where experienced nurses provide guidance to the newer nurses can enhance job satisfaction and resilience. While financial compensation improvement may be difficult in low-income countries, offering non-monetary incentives such as recognition programs and professional growth pathways can enhance job satisfaction and mental wellbeing. These interventions can collectively create a supportive environment that can result in better mental outcomes for pediatric nurses and improved care for pediatric patients. Future research should prioritize longitudinal studies focusing on evaluating the long-term effectiveness of these interventions to develop evidence based strategies for sustaining mental health improvement among pediatric nurses.

## Conclusion

In conclusion, this systematic review identifies high workload, poor work environment, limited resources, strained interpersonal relationships, lack of support, irregular shift patterns, demanding roles, and financial strain as key factors impacting the mental wellbeing of pediatric nurses. These factors interact to exacerbate stress, burnout, anxiety, and depression posing a significant challenge to both nurses' wellbeing and patients' care quality. To mitigate these challenges, pediatric units should implement evidence based interventions including manageable nurse-to-patient ratios, adequate resource allocation, fostering a supportive work culture, flexible scheduling, targeted support for senior nurses, and improved financial compensation. To ensure the long-term effectiveness of these interventions, healthcare institutions should develop policies that promote continuous monitoring and evaluation of these strategies. However, beyond implementation of these interventions, continuous monitoring of pediatric nurses' mental health is crucial. Pediatric unit administrators should institutionalize routine mental health assessments of pediatric nurses using standardized screening tools. By institutionalizing these processes, pediatric healthcare facilities can create a sustainable framework for not only improving the mental health and wellbeing of pediatric nurses, but also enhancing patient care outcomes.
